# Discordance between GCIG CA-125 progression and RECIST progression in the CALYPSO trial of patients with platinum-sensitive recurrent ovarian cancer

**DOI:** 10.1038/s41416-023-02528-z

**Published:** 2023-12-14

**Authors:** Danka Sinikovic Zebic, Angelina Tjokrowidjaja, Katherine Elizabeth Francis, Michael Friedlander, Val Gebski, Alain Lortholary, Florence Joly, Annette Hasenburg, Mansoor Mirza, Ursula Denison, Sabrina Chiara Cecere, Annamaria Ferrero, Eric Pujade-Lauraine, Chee Khoon Lee

**Affiliations:** 1 National Health and Medical Research Council Clinical Trials Centre, The University of Sydney, Sydney, NSW 2050 Australia; 2 Department of Medical Oncology, St George Hospital, Kogarah, NSW 2217 Australia; 3 Department of Medical Oncology, South East Regional Hospital, Bega, NSW 2550 Australia; 4 Department of Medical Oncology, Prince of Wales Hospital, Randwick, NSW 2031 Australia; 5 Hôpital Privé du Confluent, Nantes and GINECO, Nantes, France; 6 Centre François Baclesse, Caen and GINECO, Caen, France; 7 Department of Gynecology and Obstetrics, University Medical Center, Mainz and AGO, Mainz, Germany; 8 Rigshospitalet-Copenhagen University Hospital, Copenhagen and NSGO, Copenhagen, Denmark; 9 Institute for gynaecological oncology und senology - Karl Landsteiner, Vienna and AGO Austria, Vienna, Austria; 10 Oncologia Clinica Sperimentale Uro-Ginecologica, Istituto Nazionale Tumori - IRCCS- Fondazione G.Pascale, Napoli and MITO Italia, Napoli, Italy; 11 Academic Division Gynaecology, Mauriziano Hospital, University of Torino, and MaNGO, Torino, Italy; 12 ARCAGY-GINECO, 8 rue Lamennais, 75008 Paris, France

**Keywords:** Ovarian cancer, Ovarian cancer

## Abstract

**Background:**

CA-125 alone is widely used to diagnose progressive disease (PD) in platinum-sensitive recurrent ovarian cancer (PSROC) on chemotherapy. However, there are increasing concerns regarding its accuracy. We assessed concordance between progression defined by CA-125 and RECIST using data from the CALYPSO trial.

**Methods:**

We computed concordance rates for PD by CA-125 and RECIST to determine the positive (PPV) and negative predictive values (NPV).

**Results:**

Of 769 (79%) evaluable participants, 387 had CA-125 PD, where only 276 had concordant RECIST PD (PPV 71%, 95% CI 67–76%). For 382 without CA-125 PD, 255 had RECIST PD but 127 did not (NPV 33%, 95% CI 29–38). There were significant differences in NPV according to baseline CA-125 (≤100 vs >100: 42% vs 25%, *P* < 0.001); non-measurable vs measurable disease (51% vs 26%, *P* < 0.001); and platinum-free-interval (>12 vs 6–12 months: 41% vs 14%, *P* < 0.001). We observed falling CA-125 levels in 78% of patients with RECIST PD and CA-125 non-PD.

**Conclusion:**

Approximately 2 in 3 women with PSROC have RECIST PD but not CA-125 PD by GCIG criteria. Monitoring CA-125 levels alone is not reliable for detecting PD. Further research is required to investigate the survival impact of local therapy in radiological detected early asymptomatic PD.

## Background

The majority of women with advanced ovarian cancer will develop disease recurrence despite surgery and platinum-based chemotherapy [1]. With a growing number of treatment options these women are living longer, despite recurrent disease. Contemporary management of platinum-sensitive recurrent ovarian cancer (PSROC) now includes maintenance therapy with anti-angiogenic agents and poly(ADP-ribose) polymerase (PARP) inhibitors, and also secondary cytoreductive surgery (SCR) in selected subsets of women [2]. Therefore, surveillance and accurate detection of cancer recurrence is important to guide timely subsequent treatment.

Clinical trials require regular radiological imaging every 2–3 months, as well as serum CA-125, to monitor patients with recurrent ovarian cancer (ROC) undergoing systemic therapy. These investigations are used to ascertain treatment response as well as diagnose disease progression (PD). Progression-free survival (PFS) is the primary endpoint in most phase 3 trials and based predominantly on Response Evaluation Criteria in Solid Tumors (RECIST) for progression. However, in clinical practice most patients with ROC receiving chemotherapy have CA-125 levels monitored at regular intervals alone and radiological imaging is prompted by CA-125 rise or symptoms suggestive of progression.

The Gynaecological Cancer InterGroup (GCIG) definition of CA-125 progression is defined as doubling in CA-125 value from the upper limit of normal, or doubling from the nadir for those with elevated baseline CA-125 levels that remain persistently elevated [3–6]. These criteria for PD were developed and validated based on trial data in patients with advanced ovarian cancer receiving first-line chemotherapy [3, 4, 7]. They have subsequently become widely adopted and used as clinical trial endpoints as well as in routine clinical practice. RECIST criteria were developed in 2000, having evolved from the original World Health Organization (WHO) guidelines [8], approximately a decade after GCIG CA-125 definition of PD was established. The validation studies of CA-125 as a marker of PD [7] were conducted in the era preceding the contemporary RECIST.

Clinical practice guidelines such as National Comprehensive Cancer Network (NCCN) and European Society for Medical Oncology (ESMO) recommend surveillance with clinical assessment and CA-125 measurements, with radiological imaging reserved for patients with symptoms or signs suggestive of recurrence and/or CA-125 rise [2, 9]. Regular CT imaging is costly and may not be readily accessible to all, hence regular monitoring of CA-125 is favoured by many over CT imaging [10, 11]. When informative, CA-125 levels tend to start rising prior to RECIST progression with a lead time of approximately 3 months, and before onset of clinical signs and/or symptoms [3, 12, 13], particularly in the subgroup of women with small volume peritoneal disease that is often undetectable on CT imaging [14]. The clinical practice guidelines are less clear on monitoring patients on maintenance therapy and recommend that local protocols should be devised with a focus on toxicity assessment and disease activity [2, 9]. Imaging should be used to monitor disease based on symptoms and CA-125 levels, or on a regular basis if CA-125 was normal at the start of treatment [9].

Despite the widespread use of CA-125 to diagnose progression in both clinical trials and routine practice, there are concerns regarding its reliability and accuracy as a surrogate for PD. For example, in the AURELIA trial of platinum-resistant ovarian cancer (PROC), more than half of the patients with RECIST PD did not have progression by GCIG CA-125 criteria for PD [15]. Similar findings were observed in patients treated with maintenance olaparib or placebo in the SOLO-2 trial [16]. These findings raise important questions regarding the concordance between CA-125 and RECIST PD which has implications for practice particularly when CT imaging is not used routinely to detect recurrent disease.

We evaluated the validity of GCIG defined CA-125 PD in women with PSROC undergoing platinum-based chemotherapy and assessed the concordance between GCIG CA-125 and RECIST PD using data from the CALYPSO trial. In addition to its large sample size, the CALYPSO trial dataset is ideal for this study because it was one of the first trials in PSROC to prospectively incorporate both RECIST and GCIG CA-125 assessments for the evaluation of PD.

## Methods

CALYPSO (NCT00538603) is a large randomized phase III non-inferiority trial (*N* = 975) of carboplatin and pegylated liposomal doxorubicin (CPLD) versus carboplatin and paclitaxel (CP) in women with PSROC [17]. The trial reported a significant PFS improvement with CPLD compared to CP (hazard ratio [HR] 0.82; 95% confidence interval [CI], 0.72–0.94; *P* = 0.005). CT imaging and CA-125 measurements were performed at baseline, then every 12 weeks for up to 2 years, then every 24 weeks until PD. The details of this trial have been previously reported [17].

In this analysis, participants were included if they had baseline CA-125 and at least another CA-125 reading beyond baseline. Participants were excluded if PD was based solely on clinical progression without CA-125 or imaging. Those who progressed at randomisation were also excluded. For each participant, we included all CA-125 values. For those with documented PD based on RECIST, the CA-125 value closest to the date of RECIST PD was assessed, and the GCIG CA125 criteria [6] for PD was applied, to classify whether these participants had concordant CA-125 PD. For those without documented RECIST PD and CA-125 PD, the CA-125 value closest to the date of last follow-up was utilised to confirm non-PD.

We computed concordance rates for PD/non-PD by GCIG CA-125 criteria and RECIST. Participants were considered to have concordant CA-125 and RECIST progression if CA-125 PD occurred within 3 months prior to or up to 14 days after documented RECIST PD. We measured concordance by determining the positive predictive value (PPV) and negative predictive value (NPV). PPV was defined as the probability of participants with CA-125 PD that also had RECIST PD, and NPV as the probability that those without CA125 PD also did not have RECIST PD.

We performed subgroup analysis according to baseline characteristics to determine whether there was any significant heterogeneity in the overall results. The subgroups evaluated were treatment arms (CP versus CPLD), presence or absence of measurable disease, size of largest measurable lesion (≤1 cm versus > 1 cm), platinum-free interval (6-12 months versus >12 months) and baseline CA-125 value (≤100 versus >100).

We also conducted several sensitivity analyses. We evaluated concordance based on a wider time interval of up to 28 days for CA-125 PD to be assessed after documented RECIST PD. We also performed landmark analyses at 9, 12 and 18-month time-points as we recognised that our primary analysis may potentially be subjected to immortal time bias [18]. For the landmark analyses, patients with PD after these time-points, either by RECIST and/or CA-125, were reclassified as non-PD.

Amongst discordant groups (RECIST PD/CA-125 non-PD or RECIST non-PD/CA-125 PD), we further subdivided these participants into (1) rising CA-125 (>50% baseline at time of RECIST PD); (2) stable CA-125 (>15% below and ≤50% above baseline); or (3) falling CA-125 (≤15% below baseline). These cutoff values were based on a prior publication [16]. The CA-125 values for the three groups were presented as scatter plots and summarised using the median and the interquartile range.

PFS according to RECIST and CA-125 was estimated using the Kaplan Meier method. Median difference in time between CA-125 and RECIST PD for each treatment arm was computed.

All analyses were exploratory and not adjusted for multiple testing. All *P* values were two-sided, with *P* < 0.05 considered to be statistically significant.

## Results

**Fig. 1:**
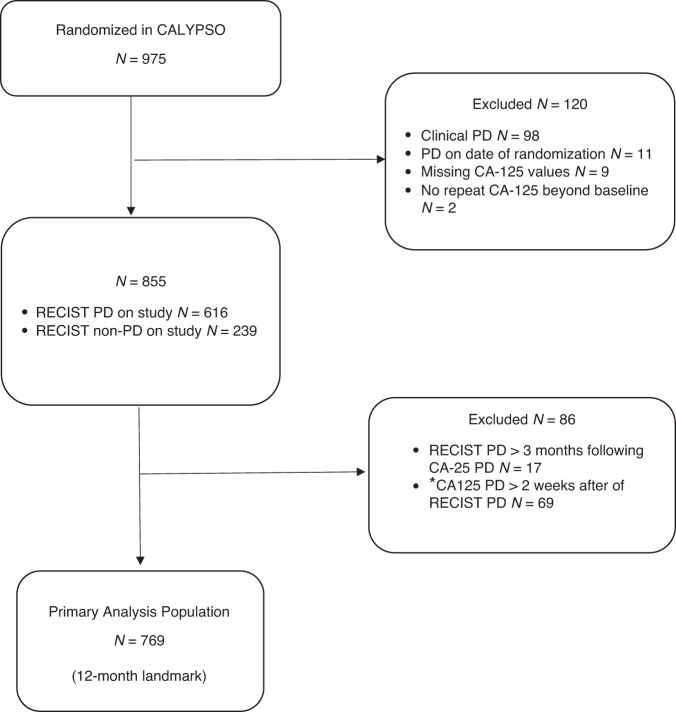
Study population flow chart. *Secondary analysis includes *n* = 804 patients with CA125 PD up to 4 weeks after RECIST PD.

Of 975 participants randomized in the CALYPSO trial, 206 (21%) were excluded (for reasons summarised in Fig. 1), leaving 769 eligible participants suitable for our primary analysis. The baseline characteristics of these participants in our primary analysis did not differ significantly from the intention-to-treat population (Table 1).

**Table 1:** Baseline characteristics.

	Analysis Population (*n* = 769)		ITT Population (*n* = 975)		
	No.	%	No.	%	*p* value
Median age, years (IQR)	61 (54–67)		61 (54–67)		0.76
Median baseline CA125, IU/L (IQR)	168 (72–458)		168 (71–458)		0.99
Treatment					
CP	399	52	509	52	0.89
CPLD	370	48	466	48	
ECOG					
0	494	64	603	62	0.82
1	241	31	322	33	
2	20	3	28	3	
Histology					
Serous	556	72	700	72	1.00
Endometrioid	56	7	73	7	
Other	157	20	170	17	
FIGO					
I–II	102	13	123	13	1.00
III	558	73	712	74	
IV	92	12	115	12	
Measurable disease					
Yes	553	72	700	72	0.96
No	216	28	275	28	
Largest tumour size					
≤1 cm	573	75	717	74	0.65
>1 cm	196	25	258	26	
PFI					
6–12 months	267	35	344	35	0.81
>12 months	502	65	631	65	
No. of previous lines of therapy					
1 line	646	84	825	85	0.67
2 lines	121	16	146	15	

*IQR* inter-quartile range, *CP* carboplatin/paclitaxel, *CPLD* carboplatin/pegylated liposomal doxorubicin, *ECOG* Eastern Cooperative Oncology Group, *FIGO* International Federation of Gynaecology and Obstetrics, *PFI* platinum free interval.

**Fig. 2:**
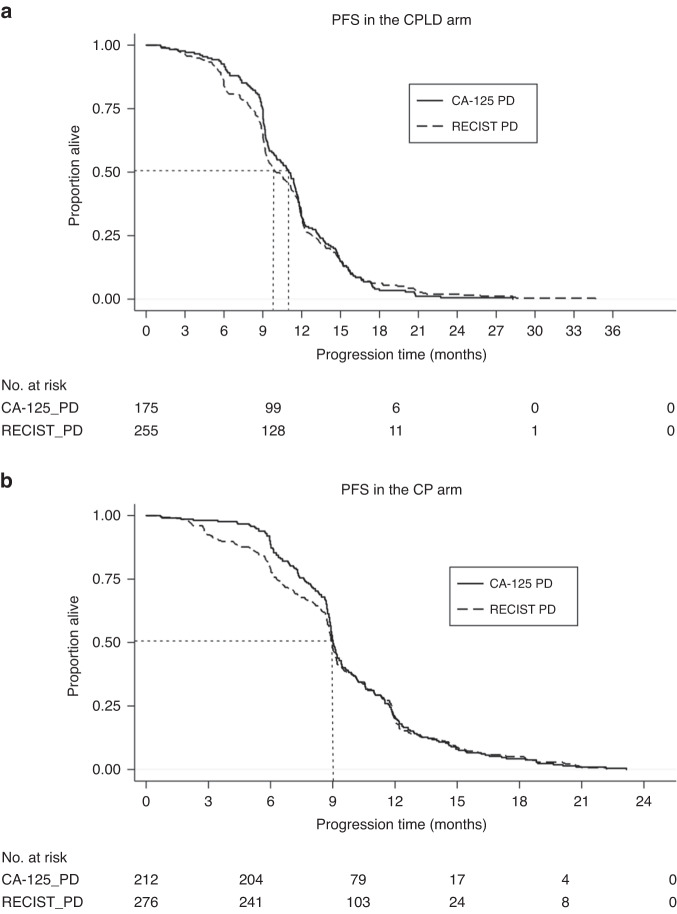
Kaplan–Meier Curve for progression free survival (PFS) in (**a**) CPLD (carboplatin/pegylated liposomal doxorubicin) and (**b**) CP (carboplatin/paclitaxel) arms. PFS progression free survival, CPLD carboplatin/pegylated liposomal doxorubicin, CP carboplatin/paclitaxel; CA-125 PD, CA-125 progression; RECIST PD, RECIST progression. *y*-axis – proportion alive, *x*-axis – progression time in months.

The median follow-up of our primary analysis population was 22 months. In the CPLD arm, the median time to PD by CA-125 and RECIST was 11 months and 10 months, respectively (Fig. 2a). In the CP arm, the median times to PD by both CA-125 and RECIST were 9 months (Fig. 2b).

### Concordance between CA-125 and RECIST assessments

**Fig. 3:**
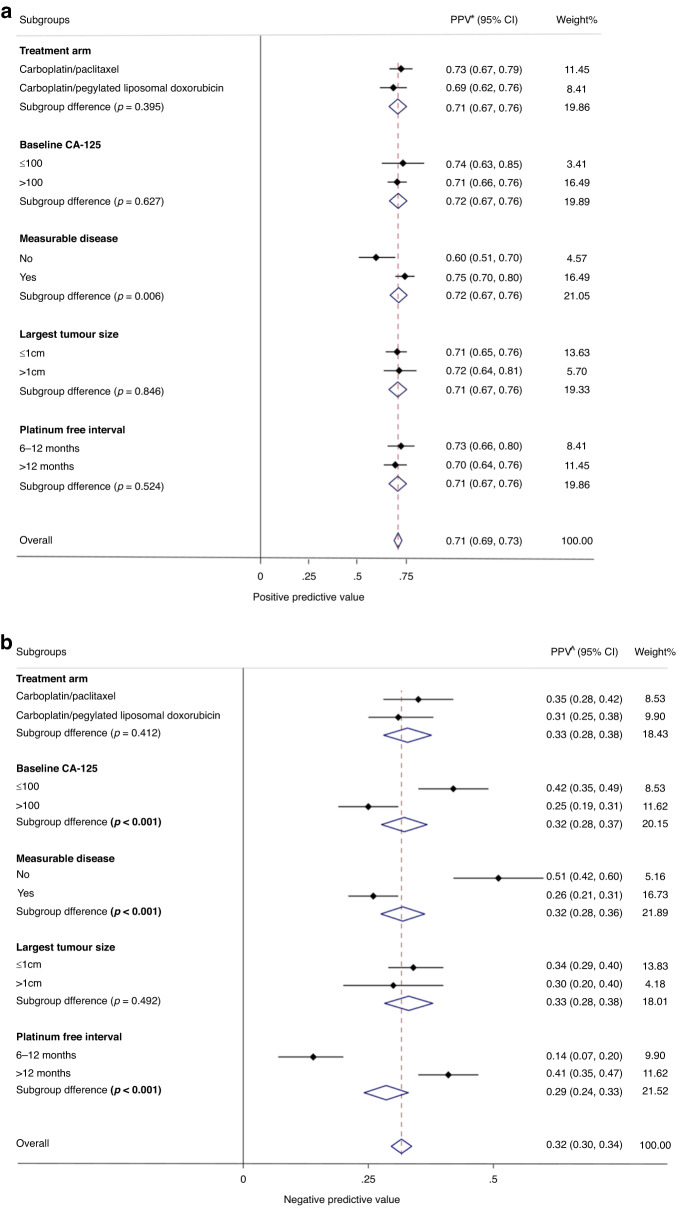
Forest plot showing (**a**) positive predictive values (PPV) and (**b**) negative predictive values (NPV) for primary analyses in patient subgroups. Concordance percentage for each subgroup variable, expressed as positive predictive value (**a**) and negative predictive value (**b**), is represented by the small diamond, and the horizontal line crossing the square represents the 95% confidence interval (CI). The large diamond represents the pooled overall measure of concordance. PPV positive predictive value (probability of patients with CA-125 PD that also had RECIST PD), NPV negative predictive value (probability that those without CA125 PD also did not have RECIST PD).

In the primary analysis population, approximately half of the participants (*n* = 387) had CA-125 PD, and of these 276 also had RECIST PD, resulting in a PPV of 71% (95% CI 67–76%) (Fig. 3a). Of those without CA-125 PD (*n* = 382), 255 had RECIST PD and 127 did not, resulting in a NPV of 33% (95% CI 29–38) (Fig. 3b).

### Subgroup analysis

Across the different subgroups of interest, there was no significant heterogeneity in the PPV (Fig. 3a). However, NPV was significantly different according to baseline CA-125 (≤100 vs >100: 42% vs 25%, *P* < 0.001; Fig. 3b). Significant differences were also seen for those with non-measurable vs measurable disease (51% vs 26%, *P* < 0.001), and platinum-free interval (>12 months vs 6–12 months: 41% vs 14%, *P* < 0.001).

### CA-125 trajectory in discordant cohorts from baseline to time of PD

**Fig. 4:**
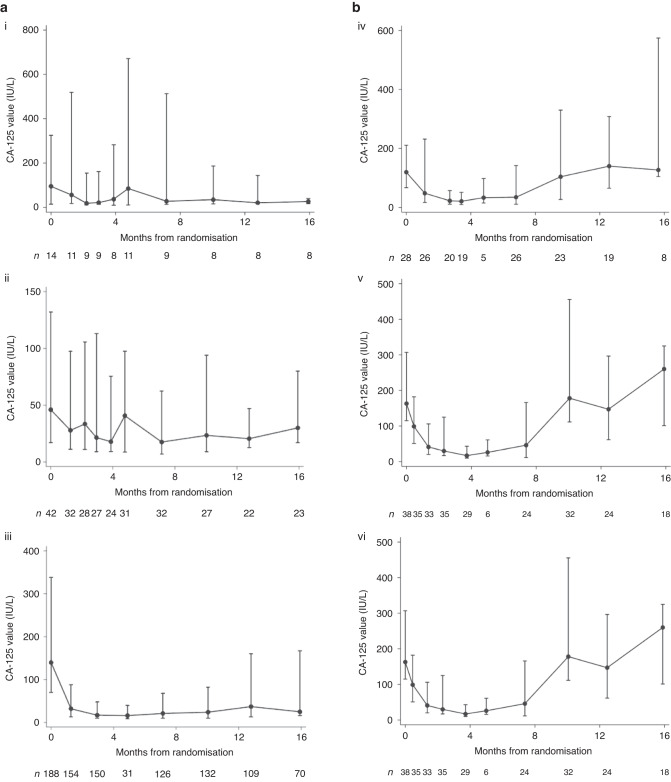
Scatter plots and median (IQR) for discordant groups: (**a**) Patients (*N* = 255) with RECIST PD only but CA-125 not meeting GCIG criteria for PD with (i) rising CA-125, (ii) stable CA-125, (iii) falling CA-125 values. **b** Patients (*N* = 111) with GCIG CA-125 PD only but without RECIST PD (iv) rising CA-125, (v) stable CA-125, (vi) falling CA-125 values. Dots represent the median CA-125 value at the particular time-point with bars representing the associated interquartile range. Patterns of CA-125 defined as: Rising CA-125 (>50% baseline at time of RECIST PD); Stable CA-125 (>15% below and ≤50% above baseline); Falling CA-125 (≤15% below baseline).

Of those with RECIST PD but CA-125 non-PD (*n* = 255), the majority (*n* = 199, 78%) had a falling trend in serial CA-125 measurements, 42 (16.5%) had stable CA-125, and only 14 (5.5%) patients had rising serial CA-125 (Fig. 4a). Of those with RECIST non-PD and GCIG CA-125 PD (*n* = 111), just over half (*n* = 64, 58%) had a falling CA-125, and 28 (25%) had stable CA-125, and 19 (17%) had rising CA-125 (Fig. 4b).

### Sensitivity analyses

When we assessed CA-125 values based on a wider time interval of up to 28 days following documented RECIST PD, the PPV (74%, 95% CI 70–78%) and NPV (33%, 95% CI 29–38%) did not differ significantly from our primary analysis (Supplementary Fig. 1). When we examined concordance using other landmark time-points, PPV was highest at the 9-month landmark (95%, 95% CI 93–97), while 12 and 18-month timepoints showed consistent results with our primary analysis (Supplementary Fig. 2A). NPV was also highest using the 9-month landmark (80%, 95%CI 75–85) and lowest at the 18-month landmark (41%, 95%CI 36-50%) (Supplementary Fig. 2B).

## Discussion

Our study demonstrates approximately 1 in 3 participants of the CALYPSO trial with GCIG CA-125 PD did not have RECIST PD, and nearly half of these had rising or stable CA-125 levels, and just over half had falling trend in CA-125 levels, from baseline to time of progression. Approximately 2 in 3 participants without GCIG CA-125 PD had RECIST PD, and the majority of those had a falling trend in CA-125 levels. This demonstrates poor reliability of CA-125 values alone as depicted with the fluctuations in CA-125 trajectory from baseline to time of PD amongst women with PSROC.

Although PPV was consistent across the different subgroups, NPV was higher among those with favourable prognostic factors, including CA-125 ≤ 100, non-measurable disease, and PFI > 12 months. This suggests that CA-125 non-PD may be more reliable in those with lower burden and/or disease that is poorly detectable by CT imaging. Despite the apparent improved performance of CA-125 non-PD in these subsets of patients, our results still show that approximately 1 in 2 women with recurrent disease would not be accurately detected with CA-125 alone. This study is also the first to evaluate the difference in discordance according to different baseline prognostic factors.

Concordance between CA-125 and RECIST PD was assessed in other trials investigating different populations of women with ovarian cancer [7, 15, 16]. The SOLO2 analysis reported a high PPV of 96% and low NPV of 52% in PSROC *BRCA*-positive women treated with or without maintenance olaparib [16]. Notably, these women had a significantly lower baseline CA-125 with median of 12 IU/L as compared to CALYPSO patients with high-volume disease and high baseline median CA-125 of 168 IU/L. Analysis of the AURELIA trial reported concordant RECIST and CA-125 PD in 43% and discordant RECIST PD and CA-125 non-PD in 57% for PROC patients treated with single-agent chemotherapy with or without bevacizumab [15]. However, the assessment of concordance was limited to only those patients with RECIST PD, with no assessment done for the RECIST non-PD population in this trial [15].

GCIG defined CA-125 criteria is widely used in clinical trials and routine practice but was based on serial CA-125 levels from the North Thames Ovary (NTO) trial nearly three decades ago with a reported NPV of 78% [7]. Progression was determined by clinical or radiological findings [19], and scan modality or frequency was not specified. Use of regular interval imaging in CALYPSO, and also improvements in the quality of CT scans [20, 21] over the past two decades may have contributed to the marked difference in NPV of CA-125 as seen in our study compared to that reported from the NTO trial.

Despite improvement of CT imaging, there are still limitations in the detection of recurrent disease in a large majority. Further, ^18^F-FDG PET/CT is increasingly being used to detect asymptomatic early disease relapse. They have become more accessible since development of GCIG criteria and RECIST and used in clinical practice but not routinely adopted in clinical trials. ^18^F-FDG PET/CT has been shown to be more sensitive and specific when compared with CT scans, 91% versus 84% and 91% versus 65% [22], respectively. Early detection of PD with ^18^F-FDG PET/CT is particularly important for consideration of SCR, since recent studies have reported improved survival when complete resection could be achieved [23, 24].

A prior *post hoc* analysis of CALYPSO found that the relative treatment effect on PFS did not differ significantly when PD was based either on CA-125 or RECIST [25]. This prior work was based on the comparison of the difference in the average treatment effect of the CP arm versus CPLD arm [25]. Our present study evaluates the data on PD differently where our focus is on the discordance in PD rates based on GCIG CA-125 versus RECIST specific to each study participant instead of relative treatment effect between treatment arms. Importantly, for each treatment arm, the time to PD by CA-125 was not identical to time to PD by RECIST. For example, in the patient cohort included to our analysis, there was a difference in the median PFS by one month for the CPLD arm when comparing PD by CA-125 with PD by RECIST (Fig. 2a). Although the median times to PD by both CA-125 and RECIST for CP arm were almost identical, differences in progression-free survival were observed for other timepoints (Fig. 2b).

Our work has several strengths. CALYPSO is a large, international randomized trial, and hence provides access to high quality CA-125 and RECIST data to perform this exploratory work. Robust findings could also be established based on important patient subgroups. There are also limitations of our work. Treatment practice has evolved since the design and conduct of CALYPSO, particularly with the introduction of maintenance therapies as well as SCR. However, the findings of our study are relevant, and are consistent with the findings of other recent publications that more reflect contemporary practice [16, 26]. CALYPSO was not designed to specifically test for the concordance of CA-125 with RECIST PD, and hence the impact on CA-125 data collection, notably confirmatory readings as mandated by GCIG criteria were absent, but in only approximately 1% of the participants. In addition, CALYPSO reported on first disease progression for the primary PFS endpoint, and subsequent data collection beyond first PD for CA-125 and RECIST was limited. Participants enrolled in CALYPSO were highly selected individuals sufficiently fit to undergo clinical trial therapies, and their outcomes were likely to be generally superior compared to those being treated in routine clinical care. Whilst we acknowledge these limitations they are not likely to impact on the applicability of our study assessing the concordance between CA-125 and radiological progression to real-world setting.

Our results have implications on routine practice particularly in the era of changing treatment paradigm for PSROC. A prior study demonstrated that initiating chemotherapy prior to development of symptoms does not impact survival [12]. However, this trial was conducted over 20 years ago and the relevance of its finding in the contemporary treatment context is questionable, where maintenance therapies are now standard of care and where SCR has an established role with improved outcomes in a selected subset of patients [23, 24, 27]. Further, maintenance therapies are costly, and it is important to cease futile treatments and evaluate alternative options promptly at disease progression. There is emerging data on the benefit of continuing PARPi maintenance beyond progression in those with oligometastatic disease that are managed with local therapies [28, 29]. According to current management guidelines where CA-125 is to be used in conjunction with clinical examination only [2, 9], based on our results approximately 50% of women who progressed will not be promptly detected with potential consequences for their subsequent treatments. We acknowledged that further research is still required to determine definitely that treatment offered to those with early asymptomatic radiological detected PD will necessarily prolong overall survival in these women.

Our results are important in guiding future research and identifying novel surrogate biomarkers in combination with or as an alternative to CA-125. KELIM, a model based on longitudinal CA-125 kinetics during initial chemotherapy treatment, has been shown to be a useful marker of tumour chemosensitivity, and has been proposed as an alternative prognostic marker of early response in place of CA-125 alone [30–33]. CA-125 KELIM has also been proposed as an independent prognostic biomarker for survival, complementary to surgery, in first-line HGSOC trials [33]. More work is required, either with the use KELIM or other similar approaches, to evaluate longitudinal CA-125 kinetics to promptly define disease progression. Other novel biomarkers, such as clearance of circulating tumour DNA and HE4 [34–37], should also be investigated to improve the accuracy of detection of cancer recurrence. Hence, review of GCIG criteria is urgently required to guide both clinical practice and design of future trials.

## Conclusion

In conclusion, we observed poor concordance between CA-125 non-PD and RECIST non-PD in women with PSROC, with 2 in 3 women without CA-125 progression having radiological progression. Our results add to the growing evidence and reinforce the importance of surveillance imaging in addition to serum measurement of CA-125 in routine practice. CA-125 alone is not reliable in detecting disease relapse and future research should focus on identifying more reliable approach of using CA-125 or other novel biomarkers that are more sensitive in detecting RECIST PD. Further research is also necessary to determine whether local therapy offered to women with early asymptomatic radiological detected PD will prolong overall survival.

### Supplementary information

Supplementary figures

## Data Availability

The data that support the findings of this study are deidentified participant data, and not publicly available but may be upon reasonable request from ARCAGY-GINECO and CALYPSO substudy steering committee.
