# Supplementary material for: Discordance between GCIG CA-125 progression and RECIST progression in the CALYPSO trial of patients with platinum-sensitive recurrent ovarian cancer
Source: Br J Cancer. 2023 Dec 14;130(3):425–33. doi: 10.1038/s41416-023-02528-z (PMC10844635; doi:10.1038/s41416-023-02528-z)
Supplement: Supplementary file 1 — Supplementary figures [file 41416_2023_2528_MOESM1_ESM.docx]

**Supplementary:**


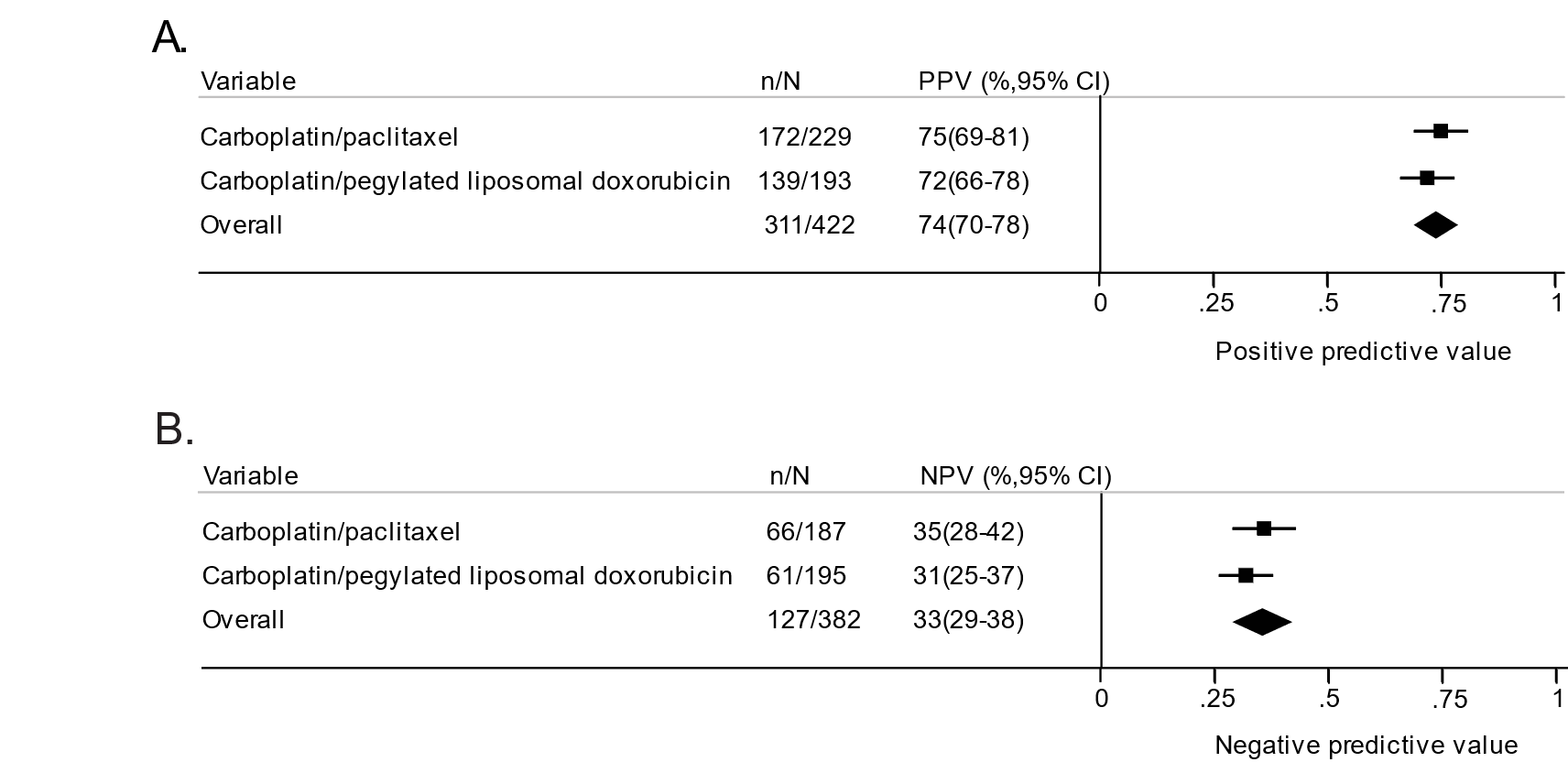
**Figure 1:** Forest plot based on 4-week window between imaging detected progressive disease and CA-125 measurement showing (A) positive predictive value and (B) negative predictive value

**Abbreviations:** PPV, positive predictive value; NPV, negative predictive value

For PPV, “n” refers to the number of patients with CA-125 PD and RECIST PD (true positive), and “N” refers to the total number of patients with CA-125 PD.

For NPV, “n” refers to the number of patients with CA-125 non-PD and RECIST non-PD (true negative) and “N” refers to the total number of patients with CA-125 non-PD.

**Figure 2:** Forest plots for (A) positive predictive value and (B) negative predictive value for landmark times at 9, 12 and 18 months


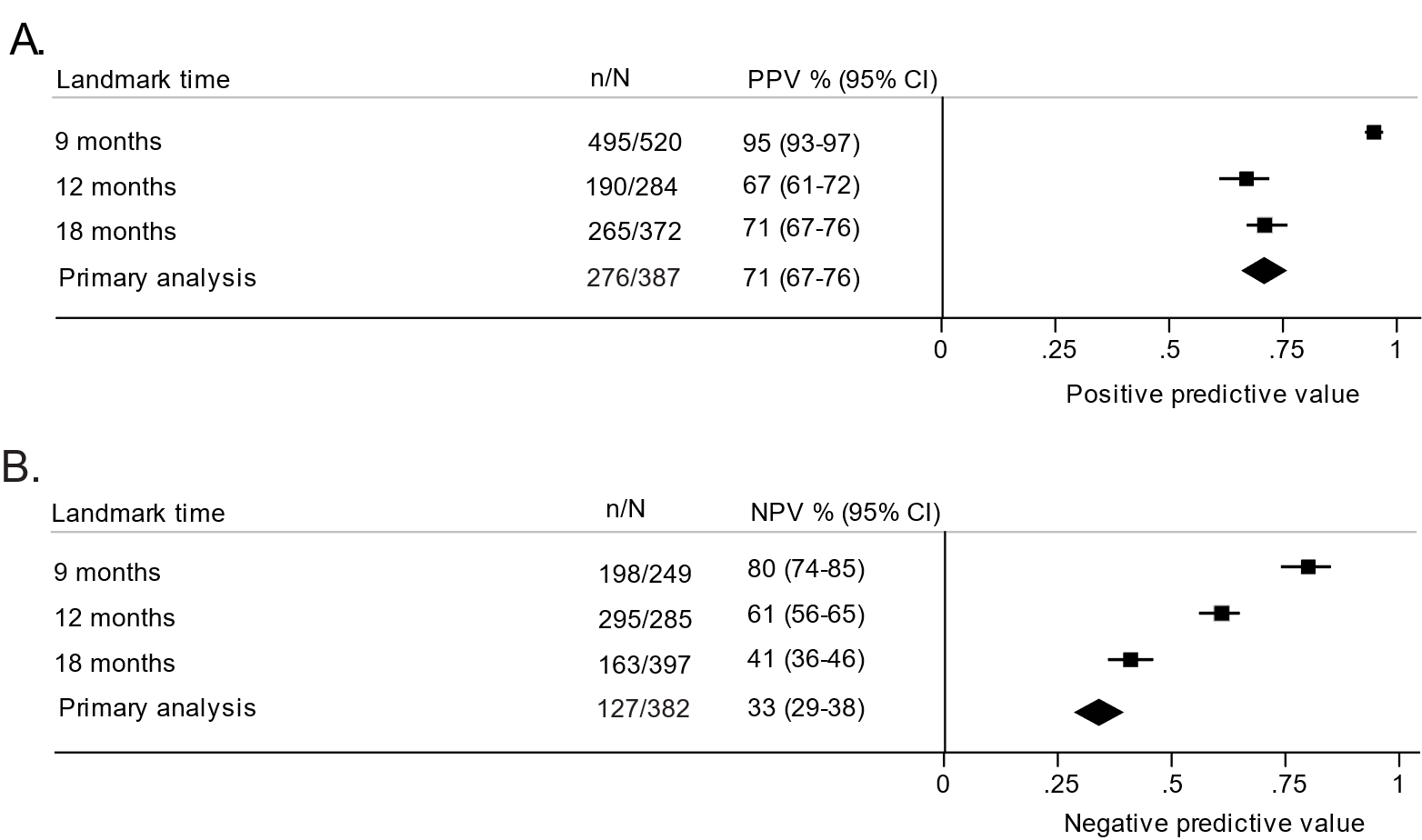


**Abbreviations:** PPV, positive predictive value; NPV, negative predictive value

For PPV, “n” refers to the number of patients with CA-125 PD and RECIST PD (true positive), and “N” refers to the total number of patients with CA-125 PD.

For NPV, “n” refers to the number of patients with CA-125 non-PD and RECIST non-PD (true negative) and “N” refers to the total number of patients with CA-125 non-PD.
